# Seventh Annual Meeting of the Tri-State Medical Society of Alabama, Georgia and Tennessee

**Published:** 1895-12

**Authors:** 


### *SOCIETY REPORTS.*

---

### SEVENTH ANNUAL MEETING OF THE TRI-STATE MEDICAL SOCIETY OF ALABAMA, GEORGIA, AND TENNESSEE.

(*Concluded from November number.*)

J. P. STEWART read a paper on "Hemorrhoids," in which he insisted on the necessity of an examination (under chloroform, if necessary). Where tumors are small he uses an injection of red gum; if larger, they are destroyed with clamp and cautery.

R. P. JOHNSON had treated cases successfully with injections of carbolic acid two parts to olive oil one part, to which was added $\frac{1}{4}$ grain morphia for each injection.

J. B. COWAN alluded to the new operation of excising all the hemorrhoidal tissue. These cases often fall into the hands of empirics, who inject carbolic acid or ergot and relieve temporarily. He likes the old operation of tying the hemorrhoids. In a bad case, where there was no distinct tumor, instead of doing the bloody operation of excising the whole tissue, he picked it up with a tenaculum and tied the membrane, thus putting in fourteen ligatures. The result was perfect.

G. A. BAXTER insisted on not confining the treatment to any one method, but varying according to the case. Carbolic acid he considered dangerous. Could not examine without paralyzing sphincter. He was always able to find a distinct tumor.

DR. STEWART said that the use of carbolic acid was a complete failure. He dissects the mucous membrane from the tumor, then puts on the clamp.

R. R. KIME read a paper on: "Synthetic Perineotomy in Lacerations of the Perineum," using the term to designate a method of dividing and dissecting without loss of tissue. The redundancy of tissue usual in these cases is due to hyperplasia, a subinvolution which will disappear when the cause is removed, viz.: the repair of the laceration. The method was described in detail. He related three cases illustrating the value of this method.

W. E. B. DAVIS read a paper on "Bile in the Peritoneal Cavity, and How to Deal with It." He presented an experimental and clinical study of the subject. His experiments confirmed the position taken in a paper read before the American Medical Association in 1892. The constant extravasation produced peritonitis, unless there was satisfactory drainage. A considerable quantity in the cavity would be walled off, just as any irritating fluid would be. It was noted that in those cases where gauze was packed around the openings in the gall-bladder or ducts that the animals recovered, as a rule. The field of operation was walled off completely. A number of animals were reopened in twenty-four to forty-eight hours and this condition found. In an operation on the human subject, in which the gall-bladder was removed and drainage with gauze and a glass tube, the field of the operation was completely walled off, and there was no evidence of general peritonitis. He took the position that, in obstruction of the common duct for a stone, an incision should be made, the obstruction removed, and drainage established, without an attempt being made to suture the opening, as these patients will not stand a lengthy operation, as a rule.

R. J. TRIPPE reported several cases of "Appendicitis," illustrating the necessity of early operation, and the fact that some will die no matter when operated on. He also gave the technique.

These two papers were discussed together.

R. R. KIME said that authors differed as to the time of operation. Many would get well anyhow, but it was difficult to tell which. Where patient was improving, and then suddenly got worse, operation is indicated, as it is probable that extravasation has taken place.

C. HOLTZCLAW had had fifteen or sixteen cases of appendicitis, but had never been called on to operate. They recovered with the use of the ice pack over the inflamed area. Operation should be performed if any evidence of suppuration, elevation of temperature, or collapse.

G. A. BAXTER said that the danger was not in operating, but in delay. The question lies in the diagnosis between catarrhal and obstructive appendicitis. When we have a distinct enlargement and induration, we have obstruction.

In a large number of *post mortems* the appendix was not found diseased one time in a hundred. Operation is indicated where there is pus, swelling, and induration.

R. H. HAYES said that in regard to appendicitis, Talamon, of Paris, had published statistics giving 90 to 95 per cent. of recoveries without operation. An eminent English authority gave out similar statistics; whether these were reliable or not he could not determine. Many abdominal surgeons claim that when any one has had an attack of appendicitis he has a constant source of danger within his belly. A few years back this source of danger was the chief indication for the operation. In this view it seemed that operation should be performed at the earliest or at any opportune time.

J. R. RATHMELL read a paper entitled, "Acromegaly: Report of a Case." The writer said that Paul Marie first described this affection in 1886, his theory being that enlargement of the pituitary gland was the cause of the enlargements which were the essential features of the disease. In the case reported, with the symptoms common to this disorder, there were two uncommon symptoms. These were long-continued, abnormal ryhthm in the respiratory act, of the Cheyne-Stokes variety, and the inability to retain either food or drink on his stomach for three months before. Both symptoms were accounted for by the enlarged pituitary body, as revealed by the *post mortem.* The gland weighed 475 grains instead of 5 to 10, in the normal condition. The writer believes the disease to be one of trophic origin, producing changes in the bony system, more particularly the bones of the face, feet, and hands; that the enlargement of the pituitary body, as well as other ductless glands, was the result and not the cause of the disease.

W. C. TOWNES read a paper on "Acromegalia," exhibiting specimens from Dr. Rathmell's case, the enlarged pituitary body and a part of the ileum with a diverticulum. He reported a case now under treatment, a marked feature of which was, that although fifty-two years old, still there was no impairment of the sexual function.

He believes the disease due to pressure on the brain due to enlargement of the pituitary body.

J. B. COWAN read a paper on "Water *versus* Atmosphere, the Cause of Malignant Malarial Fever." He related observations where water from clear springs was clearly shown to be the cause of malarial fever. One case, where two springs were connected by an underground passage, the upper spring was healthful, while those who used the lower spring had the disease. There was a marsh between the two, which formed a nidus for the development of the miasm. In almost every instance where the use of well and spring water had been abandoned for cistern water, malarial fever had disappeared entirely. In a village where all used well or spring water except one family, it alone escaped the disease. So many similar instances had come under his observation, that he invariably changed the water in his malarial cases. If sterilized water were used in malarial districts, malarial fever would be almost unknown.

Y. L. ABERNATHY observed that when we have a wet summer, that we do not have as much malarial fever. The paper seems conclusive that we can get the fever in drinking water, but why don't we have it all the year, and why don't we have it every summer? The old theory was that it was due to heat, moisture, and decomposition. Bowling's theory was that it was due to water so confined as to prevent evaporation; the modern theory is that it is due to a germ. Whichever is correct, it is true that nineteen-twentieths of the diseases of the West and South are due to malaria, so that we have to give quinine, arsenic, iron, mercury, etc.

DR. COWAN said that the reason that we did not have the disease every summer was because the heat did not develop the germ. All cases of malignant malarial fever were due to drinking-water. In parts of the South, where it was formerly prevalent and now unknown, it was due to the use of cisterns. In a case where a party had camped several years on a lake and had malaria, they escaped by taking water with them, although sleeping in the open air as before.

E. T. CAMP read a paper on "A Complicated Case of Obstetrics, with Rupture of the Uterus." There was a transverse presentation. The child was asphyxiated but resuscitated. The placenta not being delivered in an hour, chloroform was administered, the hand introduced into the uterus, when the rupture was discovered. The placenta was adherent but was torn away; morphine administered, but death took place in four hours. He thought rupture took place before the version and was spontaneous. In answer to the question as to whether it was necessarily fatal, he said that if the placenta could have been gotten away and contraction induced, the patient might have been saved.

R. H. Hayes read a paper on "The Nucleins and Their Relative Position in Sero-Therapeutics." The nucleins are the protoplastic or bioplastic cell substance. The bioplastic, primal unit of the organism; the cell life, vital and persistent force; a proteid, granular cell life substance in which all vital energy and cell life resistant force, and through which all animal nutrition took place. The nucleins were proteid bodies residing in the tissue cells and blood corpuscles, and the yeast of certain plants (animal and yeast nucleins). The former taken from the blood and lymphoid glands of the body, residing principally in the polynuclear blood corpuscles or leucocytes, the proliferation of which they have the power of increasing (leucocytosis). They are natural defenders, arresting and overwhelming all alien or disease germs as they enter the blood stream. Messrs. Vaughan and McClintock, principally the former, have developed them. They are gotten up in three forms: nuclein solution from the yeast of certain plants, vegetables; nuclein solution from tissues of the body, thymus, thyroid, liver, spleen, etc., animal; and from the tissues direct protonuclein. The principal difference between the antitoxin toxins is that the toxins antagonize or antidote a poison or ptomaine formed by the presence of alien or disease germs, and they belong to the class of albumin serums, while the nucleins belong to the alexin class of serums, and attack the germs direct as soon as they reach the blood current. The nucleins are more direct if less powerful, and have the advantage of attacking through the leucocytes any and all germs or poisons entering the system. Reports are encouraging from eminent men. The author reported an ulcer of sixteen years' standing cured in four months. Another case of ulcer of the ankle-joint (both non-tubercular) very greatly relieved in same time. He favors, from limited experience, a more general application of the nucleins.

R. P. JOHNSON read a paper on "Treatment of Diphtheria." He gave his experience with established methods, indorsing especially the benzoate of soda. He read a letter from Prof. Klebs, in which he did speak favorably of antitoxin, and claimed better results from antidiphtherine. He presented a plan of steaming the patient with quick lime by covering him with a sheet to loosen the membrane.

J. BERRIEN LINDSLEY presented a paper on the "Prevention of Smallpox." He did not believe one-fourth of the people of Tennessee were vaccinated. Doctors were not willing to admit their inability to diagnose smallpox, hence mistakes are made. In Little Rock the disease was under way six weeks before its nature was discovered. A rule of sanitary boards was, in case of doubt, to give the public the benefit and isolate the case. All suspicious cases should be sent to some institution. He related several cases where undiagnosed smallpox spread the disease, resulting sometimes in death.

The following are the officers for the ensuing year:

President, J. B. Murfree, Murfreesboro, Tenn.

Vice-Presidents, R. J. Trippe, Chattanooga; R. R. Kime, Atlanta; R. H. Hayes, Union Springs, Ala.

Secretaries, Frank Trester Smith, Chattanooga; Geo. West, Chattanooga.

The next meeting will be held in Nashville, October 13, 14 and 15, 1896.

